# Supplementary material for: Does Place Matter? An International Comparison of Early Childhood Development Outcomes between the Metropolitan Areas of Melbourne, Australia and Montreal, Canada
Source: Int J Environ Res Public Health. 2019 Aug 14;16(16):2915. doi: 10.3390/ijerph16162915 (PMC6720425; doi:10.3390/ijerph16162915)
Supplement: Supplementary file 1 [file ijerph-16-02915-s001.pdf]

Table S1. By-domain Proportions of developmentally vulnerable children among subgroups in Montreal (MTL) and Melbourne (MEL).

	**PHYS**		**SOC**		**EMOT**		**COGN**		**COMM**	
	MTL	**MEL**	**MTL**	**MEL**	**MTL**	**MEL**	**MTL**	**MEL**	**MTL**	**MEL**
Sex										
Girls	5.3% (5.0-5.7)	5.1% (4.8-5.4)	5.7% (5.4-6.1)	4.9% (4.6-5.2)	5.5% (5.1-5.9)	3.0% (2.8-3.3)	10.5% (10.0-11.0)	4.3% (4.1-4.6)	6.7% (6.2-7.1)	6.2% (5.9-6.5)
Boys	9.3% (8.8-9.8)	9.7% (9.4-10.0)	14.1% (13.5-14.7)	11.0% (10.6-11.3)	17.6% (17.0-18.3)	10.8% (10.4-11.2)	15.5% (14.9-16.1)	7.2% (6.9-7.5)	11.1% (10.6-11.6)	9.8% (9.5-10.2)
RR	1.74 (1.59-1.90)	1.92 (1.79-2.04)	2.46 (2.27-2.66)	2.23 (2.09-2.38)	3.21 (2.96-3.47)	3.55 (3.28-3.84)	1.47 (1.38-1.57)	1.68 (1.56-1.81)	1.67 (1.54-1.81)	1.59 (1.50-1.69)
RD	3.9% (3.3-4.5)	4.6% (4.2-5.1)	**8.4% (7.7-9.1)**	**6.1% (5.6-6.5)**	**12.1% (11.4-12.9)**	**7.8% (7.3-8.2)**	**5.0% (4.2-5.8)**	**2.9% (2.5-3.3)**	4.4% (3.8-5.1)	3.7% (3.2-4.1)
Home language										
French	6.4% (6.0-6.8)	---	9.0% (8.6-9.4)	---	11.5%(11.0-12.0)	---	11.3% (10.8-11.8)	---	4.6%(4.3-4.9)	---
English	8.5% (7.7-9.4)	6.9% (6.7-7.2)	11.4% (10.5-12.4)	7.1% (6.8-7.3)	11.5%(10.5-12.5)	6.6 (6.3-6.8)	13.8% (12.8-14.9)	4.7% (4.5-4.9)	13.3%(12.3-14.4)	4.8% (4.6-5.0)
Other	9.0% (8.3-9.8)	8.9% (8.4-9.4)	11.3% (10.5-12.1)	10.7% (10.2-11.2)	11.7% (10.9-12.5)	8.0% (7.6-8.5)	16.9% (16.0-17.9)	9.0% (8.5-9.5)	17.2%(16.3-18.2)	17.9% (17.2-18.6)
*RR	1.41 (1.28-1.56)	1.29 (1.21-1.38)	1.26 (1.15-1.37)	1.52 (1.42-1.61)	**1.02 (0.94-1.11)**	**1.22 (1.14-1.31)**	**1.50 (1.40-1.61)**	**1.90 (1.77-2.04)**	3.74 (3.42-4.09)	3.72 (3.51-3.94)
*RD	2.6% (1.8-3.4)	2.0% (1.4-2.6)	2.3% (1.39-3.20)	3.6% (3.0-4.2)	0.2% (-0.7-1.2)	1.5% (0.9-2.0)	**5.6% (4.6-6.7)**	**4.2% (3.7-4.8)**	12.6% (11.6-13.6)	13.1% (12.4-13.8)
Country of birth										
Home country	6.8% (6.5-7.2)	7.4% (7.2-7.6)	9.5% (9.1-9.9)	7.8% (7.6-8.0)	11.3% (10.9-11.7)	6.9% (6.7-7.1)	12.2% (11.8-12.6)	5.5% (5.3-5.8)	7.6% (7.3-7.9)	7.1% (6.9-7.3)
Outside home country	10.2% (9.1-11.5)	7.5% (6.8-8.3)	12.0% (10.8-13.4)	9.6% (8.8-10.5)	13.0% (11.7-14.4)	7.4% (6.6-8.2)	16.9% (15.5-18.5)	8.1% (7.3-8.9)	15.9%(14.5-17.4)	17.8% (16.7-18.9)
RR	**1.49 (1.31-1.70)**	**1.01 (0.91-1.13)**	1.27 (1.13-1.42)	1.23 (1.12-1.36)	1.15 (1.03-1.28)	1.07 (0.96-1.19)	1.39 (1.26-1.53)	1.46 (1.31-1.63)	**2.09 (1.89-2.32)**	**2.50 (2.33-2.69)**
RD	**3.4% (2.1-4.6)**	**0.1% (-0.1-0.1)**	2.5% (1.2-3.9)	1.8% (0.9-2.7)	1.7% (0.3-3.1)	0.5% (-0.3-1.3)	**4.7% (3.2-6.3)**	**2.6% (1.7-3.4)**	8.3% (6.8-9.8)	10.7% (9.5-11.8)
Area-level deprivation										
**Q1	4.5% (4.0-5.0)	4.3% (4.0-4.7)	7.1% (6.6-7.8)	4.6% (4.3-5.0)	9.2% (8.6-9.9)	4.8% (4.5-5.2)	9.6%(8.9-10.3)	2.2% (1.9-2.5)	5.8%(5.3-6.4)	3.5% (3.2-3.9)
Q2	6.5% (5.9-7.2)	5.5% (5.1-6.0)	8.8% (8.1-9.6)	6.4% (5.9-6.9)	10.2% (9.5-11.1)	5.8% (5.4-6.2)	11.8%(10.9-12.6)	3.9% (3.5-4.2)	7.9%(7.2-8.6)	5.3% (4.9-5.7)
Q3	8.0% (7.2-8.8)	6.9% (6.5-7.5)	10.6% (9.7-11.5)	7.4% (6.9-7.9)	12.6% (11.6-13.6)	6.6% (6.2-7.1)	13.4% (12.5-14.5)	5.4% (5.0-5.9)	9.3%(8.4-10.2)	7.0% (6.6-7.6)
Q4	8.3% (7.6-9.2)	8.7% (8.1-9.3)	11.6% (10.7-12.6)	9.7% (9.1-10.4)	12.7% (11.7-13.7)	8.0% (7.4-8.5)	14.7%(13.7-15.8)	7.5% (7.0-8.1)	10.3%(9.4-11.2)	9.9% (9.3-10.5)
Q5	10.6% (9.8-11.5)	13.4% (12.7-14.1)	13.1% (12.2-14.0)	13.3% (12.6-14.1)	14.6%(13.7-15.6)	10.7% (10.1-11.4)	17.2%(16.2-18.3)	11.8% (11.1-12.5)	12.6%(11.8-13.5)	17.2% (16.4-18.0)
**RR	**2.37 (2.07-2.70)**	**3.10 (2.81-3.43)**	**1.84 (1.65-2.05)**	**2.9 (2.6-3.2)**	**1.58 (1.44-1.75)**	**2.22 (2.00-2.45)**	**1.79 (1.64-1.97)**	**5.38 (4.71-6.15)**	**2.18 (1.94-2.46)**	**4.89 (4.41-5.44)**
**RD	**6.1% (5.16-7.10)**	**9.1% (8.3-9.9)**	6.0% (4.88-7.04)	8.7% (7.9-9.5)	5.4% (4.22-6.54)	5.9% (5.1-6.7)	7.6% (6.41-8.85)	9.6% (8.8-10.3)	**6.8% (5.79-7.89)**	**13.7% (12.8-14.5)**

(95% CIs) RR: Relative risk. RD: Risk Difference.

*For Montreal: French vs Other. For Melbourne: English vs Other. ** Q1: least deprived quintile Q5: most deprived quintile. RR and RD were calculated using Q1 vs Q5.

Bold: statistically significant difference in RR or RD between MTL and MEL.
